# Aging With Intellectual and Developmental Disabilities: A Critical Examination of Supports for Healthy Aging

**DOI:** 10.1093/geroni/igab046.866

**Published:** 2021-12-17

**Authors:** Lieke van Heumen, Kelly Munly, Patricia Heyn

## Abstract

The number of older adults with intellectual and developmental disabilities (IDD) in the U.S. is expected to double and potentially triple by 2030. Despite this demographic urgency, there continues to be a lack of research directly addressing aging of people with IDD. Individuals with IDD have on average twice as many health problems than others without IDD, experience earlier age-related declines in health and function than the general population and are more likely to develop secondary conditions as they age. The increase in the number of people aging with IDD and the challenges experienced by this population have demanded new directions for research, practice and policy that promote social justice and improve this population’s health and well-being. This symposium brings together research that critically examines and calls for a “new normal” of supports for healthy aging provided to the population aging with IDD. The first presentation consists of a systematic review of healthy aging interventions for adults with IDD. The authors conclude that such interventions for adults with IDD remain scarce, incipient and sporadic. The second presentation critically reviews an interprofessional education model aimed to address the complex and unique needs of older adults with IDD and dementia. The authors provide recommendations for the future development of interprofessional education in this field. In the third and final presentation the authors offer further transformation toward a new normal as they outline future directions for research on aging with IDD that is informed by positive psychology and disability studies theory.

